# The Manchester Conference of 1911

**Published:** 1921-07-02

**Authors:** 


### The Manchester Conference of 1911.

### RECOLLECTIONS BY A FORMER VISITOR.

The conference of the British Hospitals Association in Manchester recalls to the present writer the similar gathering which he attended ten years ago in the same city. Much water has run under the bridge since then, and certain figures then prominent and inspiring are no longer with us. The late Mr. W. G. Carnt, Superintendent of the Manchester Royal Infirmary, was the unofficial and genial host of the last occasion, and one of the objects of the conference was to inspect the great new pavilions that had only lately been completed. The new infirmary, save that it was conveniently placed within the boundaries, resembled a hospital city, and the best part of a memorable day was spent exploring the spreading pavilions and the long connecting corridors. We wanted to know how Mr. Carnt managed to administer his great institution, and floors and equipment were carefully inspected as the latest examples to be found. Mr. Carnt was ready with information, and the writer remembers him the centre of a circle of questioners dispensing replies and hospitality at the end of the day in his private room. The late Sir Henry Burdett was among those present, and with characteristic thoroughness spent his days inspecting the institutions of the city and the neighbourhood, and his nights conversing with their administrative chiefs. An institution which stands very clearly in the present writer's recollections is the Children's Hospital at Pendlebury, of which Mr. Eason was then the superintendent, before London claimed him for the Lock Hospital there. It was visited as a model children's hospital, and on the day of the writer's visit well illustrated the famous test that a well administered hospital for children is one in which no crying is heard in the wards. The quiet, harmonious colouring of the latter is a pleasant recollection, and the institution is remembered as one of the most cheerful ever entered on many visits, both before and since.

In the impressions that remain of this conference it may be said at once that the papers read and the speeches made are not part. The impression that does remain centres in the institutions visited, the friends made, and the information gathered in private talk. This fact, which must be the common experience, justifies the social side of these conferences, and the writer's recollection of a trip paid, he thinks, to the Manchester Ship Canal, is filled not with the sights of the excursion, but with the conversation on hospital topics that it engendered. And if the writer were asked how to make the best use of the present conference, he would say : Spend half your free time in the institutions, but the better half in conversation with the other members on those excursions which are chosen because they provide the most convenient opportunities for uninterrupted private talk. Mr. Frank Hazell, the present superintendent of the Royal Infirmary, though he was not at Manchester during the difficult time of transition from the old to the new institution, has been there long enough to learn the peculiarities of the Manchester hospital problem, and should be able to enlighten the conference on the difficulties of co-ordination from some recent schemes attempted there. But the writer's recollections must not run into prophecy, except to say that he is sure that Mr. Hazell will do the honours of the occasion no less hospitably than the late Mr. Carnt did ten years ago.

